# The Relationship between Workplace Conflicts and Subsequent Physician-Certified Sick Leave: A Prospective Population Study

**DOI:** 10.3390/ijerph19106047

**Published:** 2022-05-16

**Authors:** Tom Sterud, Andrea R. Marti, Eirik Degerud

**Affiliations:** National Institute of Occupational Health, 0306 Oslo, Norway; andrea.rorvik.marti@stami.no (A.R.M.); eirik.degerud@stami.no (E.D.)

**Keywords:** sickness absence, workplace conflicts, psychosocial working conditions, occupational health

## Abstract

The impact of workplace conflicts on sick leave is largely unknown. We studied the associations between conflicts and physician-certified sick leave in a randomly drawn general working population sample. Eligible respondents were interviewed in 2009, 2013, and 2016 and were registered with an employee relationship ≥50 working days in the national sick-leave register the year following the survey interviews (*n* = 22,088 observations/13,731 respondents). We used mixed-effects logistic regression models (adjusted for sex, age, education level, occupation and sick leave days) to assess the associations of self-reported conflicts with superiors or colleagues and subsequent physician-certified sick leave of 1–16 days (i.e., low-level sick leave (LLSL)) and more than 16 days (i.e., high-level sick leave (HLSL)). Conflicts with superiors were associated with LLSL (OR = 1.73 95% CI 1.15–2.62) and HLSL (OR = 1.84 95% CI 1.15–2.94). The corresponding ORs for conflicts involving colleagues were weaker and largely non-significant. The population risks of LLSL and HLSL attributable to conflicts with superiors were 1.95% (95% CI 0.55–3.41) and 3.98% (95% CI 2.08–5.91), respectively. Conflicts with superiors appear to be an important risk factor for sick leave among employees. Organizations are well-advised to develop policies and competencies to prevent and manage conflicts at work.

## 1. Introduction

Workplace conflicts are commonly defined as interpersonal interactions characterized by tension between colleagues or between superiors and employees due to ongoing or unresolved differences that can be real or perceived [1]. Interpersonal differences triggered by organizational factors such as lack of resources, competition, or poor communication more readily give rise to what has been called task-related conflicts, whereas interpersonal differences related to values, different worldviews or personality differences more readily give rise to what has been called relationship conflicts [2]. However, real-life conflicts often involve any possible combination of these issues [3]. Therefore, studies on workplace conflict as a stressor have largely conceptualized conflicts as a general measure of interpersonal conflict [4].

More than 20 years ago, interpersonal conflicts were discussed as one of the most important stressors in the workplace that can negatively affect employees’ physical and mental health [4]. In the subsequent period, research on the relationship between potentially stressful psychosocial work characteristics and stress-related disorders and sick leave has been carried out in large part within the theoretical framework of a few theoretical models: the demand–control models [5] and the effort–reward imbalance model [6] and the Job demands–resources model [7]. These models have been critical in the development of hypotheses and the generation of the current state of knowledge and have established that psychological and social stressor factors can contribute to health, disease, and absence from work [8,9,10,11]. However, a consequence of this model dominance is a lower level of evidence for many specific stressors that are not explicitly defined as part of a model, but can nevertheless be defined as job stressors that can have a bearing on employee health and be applicable to interventions [8,12]. Workplace conflicts that pose a threat to one’s interests, identity, goals, beliefs or activities could, according to the theory of ‘stress-as-offense-to-self’, be experienced as a threat to one’s self-esteem and provoke attempts to improve and protect a positive self-view that can entail significant cost for employees and induce stress-related emotional reactions [13]. According to this perspective, workplace conflicts can be hypothesized to act as any other chronic stressor that can increase the risk of a stress-related health condition and consequently influence sickness absence from work [3,4].

However, to date, the consequences related to stress from workplace conflicts have been elucidated in a relatively small number of prospectively designed studies. A study of a sample of the working population in the Netherlands reported supervisor conflict as a risk factor for the onset of prolonged fatigue and poor self-reported health [14]. Studies of the working population of Sweden [15] and Norway [16] report workplace conflicts as a risk factor for psychological distress. Several studies of the Finnish working population report interpersonal conflicts at work as a risk factor for physician-diagnosed mental disorders [17], heavy alcohol consumption and use of tranquilizers among men [18,19] and work disability among women [20]. In contrast, a study of the German working population reported that workplace conflicts, evaluated at the occupational level, were not associated with depression symptoms [21]. Furthermore, although stress-related disorders are among the leading causes of long-term sickness absence [22], studies that shed light on whether workplace conflicts affect the level of sickness absence among employees are in short supply. We identified only one cross-sectional study of a general working population in Latvia that has reported associations between managers–employee conflicts and self-reported medically certified absence [23].

The present study aimed to fill this gap in the literature by investigating possible associations between self-reported workplace conflicts and the risk of subsequent physician-certified sick leave in a representative sample of the general working population. More specifically, our objective was to address the following research questions.

R1: Does the frequency of employee conflicts with colleagues increase physician-certified sick leave?
R2: Does the frequency of employee conflicts with superiors increase physician-certified sick leave?
R3: What is the importance of workplace conflicts as a risk factor for sick leave at the population level (i.e., the population attributable risk)?

## 2. Materials and Methods

### 2.1. Study Design and Study Population

The Survey of Level of Living-Working Conditions is an ongoing representative survey of Norwegian residents aged 18–66 years, where Statistics Norway collects data every three years, predominantly by personal telephone interview (0.5% of the interviews were completed face-to-face). The survey was carried out by Statistics Norway according to the statutory rules. Statistics Norway has appointed its own privacy ombudsman, approved by the Norwegian Data Inspectorate. All persons gave their informed consent prior to their inclusion in the study. The design of the study includes a longitudinal element (that is, individuals are invited to participate multiple times) and the present study included data from three consecutive surveys. The first survey (data collection: June—10 January 2009) conducted 12,255 interviews (60.9%) out of a gross sample of 20,136 randomly drawn from the population. The second survey (April—14 January 2013) invited the same gross sample to participate and 10,875 responded (53.1%). In the third survey (September—17 April 2016) only two-thirds of the original gross sample was re-invited, and one-third was replaced with a new randomized subsample due to a planned rotation of the panel selection. In total, 10,655 interviews (52.6%) (see Table 1 for a full sample description). Statistics Norway reports few differences between the respondents and the gross sample on the benchmarks of age, sex and region [24]. From the three surveys, we included only respondents who reported to be in paid work for at least one hour or temporarily absent from work during interview week. Furthermore, the respondent had to be registered with an employee relationship of at least 50 working days in the year of the survey, as well as in the subsequent year, as judged by data from the Norwegian Labor and Welfare Administration’s sickness benefit register. Respondents that were self-employed with no employees, had missing values on either of the exposure variables, education level or occupation were excluded.

### 2.2. Measurements

#### 2.2.1. Outcome

Data on sick leave were available as the accumulated number of physician-certified sick leave days during a calendar year (that is, 2010, 2014 and 2017 constitute the follow-up period for each of the surveys). Due to the skewness and clustering around zero, the variable was recoded into three categories: ‘0 days’, ‘1–16 days’ (low level of sick leave, (LLSL)) and ‘>16 days’ (high level of sick leave (HLSL)). The cut-off (16 days) was chosen for two reasons: (1) it distinguishes between sick absence days paid by the employer and sickness absence days paid by the Norwegian Labor and Welfare Administration, and (2) it was close to the median number of sick leave days among respondents with sick leave (that is, 15 days). In Norway, employees receive full compensation from the first day of sick leave. Employees have the right to self-certification for three sick leave periods of up to three days, and some up to eight consecutive days. If a single period of absence exceeds the specified number of days (i.e., 3 or 8 days), a physician’s certificate is required.

#### 2.2.2. Exposure

The survey questionnaires were originally developed by a Nordic expert group [25], and have later been slightly modified. Workplace conflicts were measured with two items developed by Statistics Norway that uses cognitive interviews to test and develop survey questions [24]. Conflict with colleagues was assessed with the question ‘At your workplace, do you find yourself involved in unpleasant conflicts with colleagues often, sometimes, seldom or never?’ Conflicts with superiors were evaluated with the question ‘At your workplace, do you find yourself involved in unpleasant conflicts with superiors often, sometimes, rarely or never?’ The same items have been reported to predict symptoms of anxiety and depression in a previous study [16].

#### 2.2.3. Covariates

Sex, age, level of education, number of actual working days and baseline sick leave were based on administrative registry data. Age and education were treated as continuous variables in the regression analyzes but recoded as dummy variables for descriptive purposes (Table 2). The occupation was based on an open questionnaire and coded into a professional title according to the International Standard Classification of Occupations (ISCO-08) by trained interviewers. We combined 1-digit and 2-digit codes and recoded them into 17 occupational groups to obtain sufficiently large groups to present data (see Table 2).

#### 2.2.4. Statistical Analyses

The distribution of exposure to workplace conflicts and sick leave by covariates was described and differences were tested using Chi-square tests. To assess the association between workplace conflicts and the risk of subsequent sick leave, we applied generalized linear mixed models (GLMM). Mixed-effects logistic regression was our preferred method, as it is appropriate when analyzing non-normal outcome variables that are clustered within units (in the current case repeated observations from the same individuals), and when the follow-up time does not vary between individuals (the follow-up period was set to one year for all individuals since information regarding precise start and stop dates for a given period of sick leave was not available). Moreover, GLMMs utilize all available data by computing maximum likelihood estimates based on valid data from at least one time point. Prospective associations were reported as odds ratios (OR) with 95% confidence intervals (CI). We set a significance level of 0.05. All analyses were carried out using the statistical software R version 4.0.2 (R Foundation for Statistical Computing, Vienna, Austria). To address the issue of possible selection bias related to missing values and attrition (i.e., lost to follow-up), we tested (i) whether missing values in any exposure variable predicted sick leave at follow-up, and (ii) whether attrition was dependent on exposure, outcome and confounder variables at baseline. In the main analyses, we computed two time-lagged regression models with sick leave regressed on the workplace conflicts measured the previous year. Both models included random intercepts to control for nonindependence of measurements within individuals (i.e., considering the level of sick leave of the individual over time). Model#1 was adjusted for sex, age, number of actual working days, and number of sick leave days during the baseline year, while additional adjustments for occupation and educational level were made in Model#2. Furthermore, workplace conflicts were analyzed both as continuous and categorical variables (to evaluate dose–response relationship estimates for all four response categories were presented). Furthermore, we dichotomized conflicts into exposed for those who answered ‘often’ or “sometimes” on either of the two questions. Finally, we calculated the population attributable risk percent (PAR %) of sick leave attributed to workplace conflicts based on the ORs of the dichotomized exposure variables (not exposed vs. exposed). Unlike OR estimates, the PAR estimates combine data on the prevalence of exposure and a measure of association to provide a quantitative estimate of the proportion of cases in the population that are attributable to a particular exposure. The PAR estimates were calculated using the formula Pd × ((OR − 1)/OR), where Pd is the proportion of cases exposed to the risk factor in question. The lower and upper limits of the 95% CI for PAR% were calculated from the general formula of PAR% using the lower and upper limits of the 97.5% CI for Pd and OR [26].

## 3. Results

### 3.1. Sample Characteristics

In total, 21,221 observations and 13,473 respondents were included in the statistical analyses. Table 2 shows the prevalence of workplace conflicts and sick leave distributed by covariates. The prevalence of conflicts (sometimes/often) was 9.0% for conflict with superiors and 8.1% for conflict with colleagues. Both types of workplace conflicts were associated with occupation and age. Sex (being female) and educational level were associated with conflicts with colleagues, but not with superiors. The prevalence of sick leave was 18.4% (*n* = 3986 observations) for LLSL and 16.1% (*n*= 3492 observations) for HLSL. Sick leave was associated with being a woman, lower education levels, and occupation. Higher age was associated with a higher prevalence of HLSL and a lower prevalence of LLSL.

### 3.2. Analyses of Missing Values and Attrition

Respondents with missing values in either of the exposure variables (*n* = 296 observations) had a significantly lower OR for both LLSL (OR = 0.52 95% CI 0.36–0.77) and HSLS (0.57 95% CI 0.36–0.90) at follow-up, compared to the sample included in the statistical analyses. Respondents who were lost to follow-up because they did not meet the inclusion criteria at follow-up (that is, registered with <50 actual working days in the sickness benefit register) constituted 3.6% of the respondents (*n* = 832 observations). Attrition was associated with HLSL (OR = 2.20 95% CI 1.81–2.86), but not with LLSL (OR = 0.97 95% CI 0.76–1.23) at baseline. Neither conflicts with colleagues nor conflicts with superiors at baseline were associated with a higher risk of attrition, except for the highest level of exposure (often) to conflicts with superiors (OR = 2.64 95% CI 1.55–4.51) (analyses not shown).

### 3.3. Main Analyses

Table 3 shows the OR with 95% CI for sick leave according to conflicts with colleagues and superiors.

Conflicts with colleagues showed weak associations with both LLSL and HLSL in model#1. In the fully adjusted model#2, the ORs increased slightly, but most estimates (19 out of 20) did not reach the set level of statistical significance. The highest ORs were observed for the highest level of exposure (often) for both LLSL (OR = 1.23 95% CI 0.69–2.20) and HLSL (OR = 1.64 95% CI 0.88–3.03). All exposure levels were in the direction of a higher risk for both LLSL and HLSL. A nonsignificant test for trend was observed for LLSL (OR = 1.06 95% CI 0.99–1.13, *p* = 0.08), while a significant test for trend was observed for HLSL (OR = 1.09 95% 1.01–1.17). PAR estimates for conflicts with colleagues were not calculated because there were no statistically significant associations with LLSL or HLSL.

Conflicts with superiors showed consistent and statistically significant associations for both LLSL and HLSL in both partially and fully adjusted models. When exposure categories were investigated separately, we observed a higher risk of sick leave for all exposure categories, except for rarely conflicts with superiors. The highest ORs were observed for the highest exposure level (often) for both LLSL (OR = 1.73 and HLSL (OR = 1.84 95% CI 1.15–2.94). A trend test was significant for conflicts with LLSL (*p* < 0.001) and HLSL (*p* < 0.001). The PAR of LLSL and HLSL attributable to conflicts with superiors were 1.95% (95% CI 0.55–3.41) and 3.98% (95% CI 2.08–5.91), respectively.

## 4. Discussion

This study investigated the role of conflicts with colleagues and superiors at the workplace as risk factors for physician-certified sick leave in the Norwegian workforce. Workplace conflicts and sick leave were related to sex, age, education level and occupation. After adjusting for these variables and baseline sick leave, we observed consistent associations between conflict with superiors and a subsequent higher level of physician-certified sick leave. However, conflicts with colleagues were not associated with the same pronounced risk of subsequent sick leave.

Our finding of a robust prospective association between conflicts with superiors and a higher risk of high-level sick leave in a representative sample of the general working population contributes to the limited literature on this topic. To our knowledge, this is the first study to examine the prospective association between workplace conflicts and physician-certified sick leave. Therefore, comparability with other studies is limited. However, a cross-sectional study of a general working population in Latvia has reported an association between manager–employee conflicts and self-reported absence [23]. Furthermore, several studies have reported prospective associations between different aspects of poor leadership (i.e., lack of support, relational injustice and lack of recognition) and a higher risk of sickness absence [27]. Thus, the present study further underlines the more general inference that the employee’s experience of managerial behavior may have an impact on sick leave. However, our results did not indicate that being involved in conflicts with colleagues was associated with the same pronounced risk of subsequent sick leave. Although the point estimates in the fully adjusted model were in the direction of a slightly higher risk of sick leave, there was considerable uncertainty regarding the risk estimates. Consistent with this, we estimate the proportion of LLSL and HLSL attributable to conflicts with superiors only, which were two and four percent, respectively. Theoretically, this can be interpreted as the proportion of sick leave in the population that may be preventable if conflicts are eliminated, but several limitations must be considered. First, this interpretation rests on the assumption that the exposure–response relationship is causal, for which there is limited evidence. Second, the accuracy of the PAR depends on the completeness of the specified model, and despite a thorough control of confounding factors in the fully adjusted model, we cannot rule out residual confounding.

The observed association between workplace conflicts with superiors and sick leave adds to the large body of studies that over the past decades have brought convincing findings that psychosocial factors at work can contribute to stress-related disorders and sick leave [8,9,10,11]. Furthermore, this association can be interpreted from the perspective of the ‘stress as offense to self’ theory [13], which postulates that serious conflicts pose a threat to one’s self-esteem. In a workplace setting, people often have high regard for their professional roles. Consequently, a threat to your professional role may present a threat to your self-esteem. Persistent or repeated conflicts with superiors can entail significant costs for employees and thwart, or threaten to thwart, important needs and goals, for example, related to opportunities for professional development and career possibilities. Thus, it seems likely that conflicts with superiors may induce a variety of stress-related emotional reactions. In the short run, conflicts can lead to feelings of frustration or anger. Over time, failure to resolve conflicts in an appropriate manner can induce feelings of psychological distress and is likely to make an individual apprehensive about coming to work. Consistent with this reasoning, previous studies have linked workplace conflicts to mental health problems [16,28,29], which is an important cause of sick leave [30,31]. In comparison, conflicts with colleagues may not, to the same extent, pose a threat to one’s professional work role, and this provides a possible explanation for the weaker and less consistent results of conflict with colleagues, compared to conflict with superiors, in the present study.

The strengths of this study are the large, nationwide random sample with an acceptable response rate, the prospective design, and the utilization of different types of measurement for exposure (i.e., self-report) and outcome (registry-based sick leave). Furthermore, the nonresponse examination by Statistics Norway showed minor differences between nonresponders and responders across the benchmarks of age, sex and geographic region, whereas nonresponse was higher among respondents with an elementary education level. The present analyses showed that HLSL and conflicts with superiors were associated with a lower probability of attrition. This may have contributed to attenuation of the association between conflict with superiors and HLSL. In general, we observed weak and inconsistent associations between both exposure and outcome variables and attrition. Thus, it is not likely that this has affected the estimates to any great extent. However, the study also has several limitations. Sick leave was measured as the accumulated number of days during a calendar year because the precise start and stop dates for a given period of sick leave were not available. Thus, theoretically, it is possible that several sick leave periods add up to our definition of HLSL. However, since employees have the right to self-certification for three sick leave periods of up to three or eight consecutive days, it is less likely that very many employees with HLSL have several short-term spells. Furthermore, exposure data were collected by self-report, and this may have influenced the results in different ways. First, the construct validity of our workplace measure has not been extensively tested and does not differentiate between interpersonal and task-related conflicts, which can have a different impact on employee stress and well-being [3,32,33]. Thus, this is an important limitation. Furthermore, we use the self-labeling method [34], which emphasizes the subjective experience of being involved in a conflict, to measure workplace conflicts. Although the self-labeling approach is common within the sociopsychological tradition, which emphasizes that subjective experience is important in predicting responses and health outcomes, it has also been criticized for introducing subjective bias [34]. As an example, in a study in which workplace conflicts were evaluated at the occupational level, no association was observed with depressive symptoms, supporting the inference that possible links between workplace conflicts and impaired mental health could be explained by subjective perceptions of conflicts as a job stressor [21]. Thus, it cannot be ruled out that some unmeasured state or trait influencing exposure and sick leave may have inflated the estimates. To reduce the risk of reporting bias and reverse causation due to sickness absence prior to the survey, we adjusted for sick leave days the year of the survey. However, although baseline adjustment may reduce these biases, it may introduce others and may lead to over-adjustment if baseline differences reflect the effect of previous exposure to workplace conflicts [35].

## 5. Conclusions

Our findings support the inference that workplace conflicts with superiors are associated with higher levels of physician-certified sick leave in the general working population. Relative risk estimates ranged from small to moderate, but given that these types of conflict are prevalent, the total impact on sick leave in the general working population is not negligible. Thus, the results indicate that workplace conflicts are potentially important and modifiable risk factors for sick leave. Therefore, early identification and routines to deal with workplace conflicts in organizations are important. However, more longitudinal studies that assess not only the immediate, but also the delayed, and the more distal consequences of conflict at work must replicate our results. Furthermore, the literature suggests that the conceptual distinction between task-related conflicts and relationship conflicts is important and future studies should consider both the frequency and severity of different types of workplace conflict to adequately assess the levels of workplace conflicts and their impact on different health outcomes. Lastly, from a public health perspective, there is a need for more research-based knowledge on whether initiatives to develop policies and competencies to prevent and manage workplace conflicts are successful in mitigating the negative impact that workplace conflicts can inflict on worker well-being and health.

## Figures and Tables

**Table 1:** Description of the sample.

	Sample per Survey			Sample in Total	
	2009	2013	2016	Observations	Individuals
Gross sample ^a^	20,136	20,492	20,272	60,900 *	
Net sample ^b^	12,255	10,875	10,665	33,795	20,341
Response percentage	60.9%	53.1%	52.6%	55.5%	
Working population ^c^	9279	8375	8329	25,983	15,866 ^d^
Active employee relationship of at least 50 days ^e^	7709	7077	7302	22,088	13,731
Eligible sample ^f^				21,221	13,473

^a^ = random-drawn population sample (* maximum number of possible observations); ^b^ = total number of respondents including employed and non-employed individuals; ^c^ = Respondents who were in paid work for at least one hour during the interview week or were temporarily absent from such work were interviewed about the working conditions; ^d^ = sum of the people who were interviewed about working conditions in one survey (*n* = 8504), two surveys (*n* = 4607) and three surveys (*n* = 2755); ^e^ = registered with an active employee relationship of at least 50 actual working days in the survey year and the following year in the sickness absence register; ^f^ = eligible sample after deletion of respondents with missing values (*n* = 258 (1.9%) individuals).

**Table 2:** Prevalence of workplace conflicts and sick leave by sex, age, education and occupation.

	*n*	Conflicts with Superiors	Conflict with Colleagues	LLSL (1–16 Days) (%)	HLSL (>16 Days) (%)
Total	21,221	9.0	8.1	18.4	16.1
Sex					
Men	10,997	8.8	7.4	15.2	12.2
Women	10,224	9.3	8.8	22.2	20.4
Chi square tests		1.6 (1) ^NS^	14.9 (1) *	544.6 (2) *	
Age groups					
17–34	5436	8.2	7.5	21.0	14.4
35–49	8297	8.9	8.7	18.0	16.2
50–66	7488	9.7	7.7	17.5	17.4
Chi square tests		8.1 (2) *	8.1 (2) *	40.9 (4) *	
Education level					
Elementary level	2429	8.0	6.7	21.2	21.9
Upper secondary education, not completed	1934	8.5	5.7	19.2	19.6
Upper secondary education	6984	9.6	8.4	19.4	17.1
University/college 4 years	6768	9.0	8.8	18.3	14.9
University/college 4 years+	3106	8.8	8.3	15.1	10.3
Chi square tests		6.8 (4) ^NS^	27.1 (4) *	250.3 (8) *	
Occupations (ISCO code)					
Managers (11–14)	2316	8,7	12,1	11,7	12,4
Professionals (21, 24–26)	3337	8.0	6.9	16.4	11.4
Health professionals (22)	1158	10.4	10.0	22.0	19.4
Teaching professionals (23)	1533	9.8	9.7	19.1	17.0
Technicians, associate professionals (31, 33–35)	3920	8.4	7.1	17.9	13.4
Health associate professionals (32)	570	12.3	9.8	20.7	21.6
Clerks (41)	692	8.1	6.5	18.4	18.6
Customer services clerks (42–44)	580	7.4	6.7	22.9	15.2
Personal service workers (51)	1378	9.6	8.5	22.9	22.6
Sales workers (52)	1030	9.5	6.0	22.7	14.1
Personal care workers (53)	987	10.2	9.4	26.5	26.3
Protective services workers (54)	91	16.5	15.4	24.2	17.6
Agricultural/forestry/fishery workers (61–64)	81	9.9	7.4	14.8	21.0
Craft and related trades (71–75)	1782	8.9	6.6	19.3	16.4
Plant-/machine operator or assembler (81–83)	1065	10.8	6.5	16.4	21.1
Elementary occupations (91–96)	414	7.2	6.3	23.4	24.4
Unspecified	287	6.3	5.2	12.5	16.0
Chi square tests		37.7 (16) *	108.5 (16) *	577.6 (32) *	

LLSL = low level of sick leave; HLSL = high level of sick leave; ISCO = international standard classification of Occupations); * = *p* < 0.05; NS = not statistically significant.

**Table 3:** Mixed-effects logistic regression: Sick leave regressed on workplace conflicts at baseline (LLSL = low level of sick leave; HLSL = high level of sick leave).

	Sick Leave ≤ 16 Days				Sick Leave > 16 Days			
	*n* * = 17,791. Cases ^†^ = 3943				*n*^‡^ = 17,278. Cases ^†^ = 3430			
	Conflict Colleagues		Conflict Superiors		Conflict Colleagues		Conflict Superiors	
	OR ^§^	95% CI	OR ^§^	95% CI	OR ^§^	95% CI	OR ^§^	95% CI
Model#1 ^¶^								
Never (ref.)	1.00		1.00		1.00		1.00	
Rarely	1.03	(0.95–1.13)	1.12	(1.03–1.23)	0.93	(0.84–1.03)	1.04	(0.94–1.15)
Sometimes	1.11	(0.95–1.31)	1.21	(1.03–1.42)	0.94	(0.78–1.13)	1.39	(1.17–1.65)
Often	0.97	(0.53–1.77)	1.73	(1.15–2.62)	1.26	(0.67–2.35)	1.76	(1.10–2.82)
Trend, continuous	1.05	(0.98–1.12)	1.14	(1.07–1.21)	1.06	(0.98–1.14)	1.19	(1.10–1.27)
Pooled estimate ^‡‡^	1.09	(0.94–1.27)	1.25	(1.08–1.45)	1.18	(0.99–1.40)	1.52	(1.29–1.78)
Model#2 ^¶¶^								
Never (ref)	1.00		1.00		1.00		1.00	
Rarely	1.05	(0.97–1.15)	1.12	(1.03–1.23)	1.03	(0.93–1.14)	1.09	(0.99–1.21)
Sometimes	1.12	(0.95–1.32)	1.21	(1.03–1.42)	1.18	(0.98–1.42)	1.52	(1.28–1.80)
Often	1.23	(0.69–2.20)	1.73	(1.15–2.62)	1.64	(0.88–3.03)	1.84	(1.15–2.94)
Trend, continuous	1.06	(0.99–1.13)	1.13	(1.06–1.20)	1.09	(1.01–1.17)	1.20	(1.12–1.28)
*Pooled estimate* ^‡‡^	1.13	(0.97–1.31)	1.26	(1.09–1.46)	1.13	(0.96–1.35)	1.47	(1.26–1.72)
*PAR%* ^‡‡‡^	na		1.95	0.55–3.41	na		3.98	2.08–5.91

Ref. = reference value; * = net sample that excludes cases (observations) of HLSL from the denominator; ^†^ = number of sick leave observations during follow-up; ^‡^ = Net sample that excludes cases (observations) with LLSL from the denominator; ^§^ = fixed effects from the random effects logistic regression models; ^¶^ = adjustment for sex, age and number of actual working days and sick leave days the year of the survey interview (continuous) + random intercept; ^¶¶^ = + occupation and education level (continuous); ^‡‡^ ‘sometimes or often’; ^‡‡‡^ PAR% = calculated population attributable risk percentage (PAR%) based on statistically significant pooled ORs from model#2. na: not available.

## Data Availability

Statistics Norway has an established policy for data sharing. Requests for data (i.e., The Norwegian Survey on Living conditions—working conditions) can be addressed to the Norwegian Centre for Research Data (https://nsd.no/).
